# Lentil Fortified Spaghetti: Technological Properties and Nutritional Characterization

**DOI:** 10.3390/foods10010004

**Published:** 2020-12-22

**Authors:** Vita Di Stefano, Antonella Pagliaro, Matteo Alessandro Del Nobile, Amalia Conte, Maria Grazia Melilli

**Affiliations:** 1 Department of Biological, Chemical, and Pharmaceutical Science and Technology (STEBICEF), University of Palermo, 90123 Palermo, Italy; vita.distefano@unipa.it; 2 CREA Council for Agricultural Research and Economics—Research Centre for Cereal and Industrial Crops of Acireale, 95024 Catania, Italy; antonella.pagliaro@crea.gov.it; 3 Department of Agricultural Sciences, Food and Environment, University of Foggia, 71122 Foggia, Italy; matteo.delnobile@unifg.it (M.A.D.N.); amalia.conte@unifg.it (A.C.); 4 Institute of BioEconomy, National Council of Research, 95126 Catania, Italy

**Keywords:** lentil, essential fatty acids, essential amino acids, carboxymethyl cellulose, durum wheat spaghetti, fortified pasta

## Abstract

Lentil (*Lens culinaris*), consumed as a part of the diet worldwide, is a functional dietary ingredient that plays a function in human nutrition as a rich source of bioactive nutrients (low quantities of fat, sodium, and vitamin K; high content of potassium, essential amino acids, insoluble dietary fiber, and polyphenols). In this study spaghetti fortified with lentil flours (40% *w*/*w*) were developed and characterized. The addition of two different lentil flours significantly affected the sensory attributes and cooking properties of dry spaghetti. Therefore, the addition of carboxymethyl cellulose was adopted as technological option to improve the quality of fortified pasta; specifically, sensory acceptability, cooking loss, swelling index, and water absorption were studied. Chemical results highlighted that the addition of lentil to semolina significantly increased the content of lysine and threonine. It was observed an increase in essential and branched-chain amino acids. Contrary to what was expected, no increase in mono and polyunsaturated fatty acids was observed in fortified spaghetti, due to their loss during cooking, even after the addition of carboxymethyl cellulose.

## 1. Introduction

Modern consumers look for products with healthy properties and are ready to pay more for foods that claim to boost health and weight loss [1]. For this reason, the diffusion of new products promoting health and wellness is highly desirable. In this context, functional foods play an exceptional role and represent one of the most exciting areas of research and technological innovation in the food sector.

Pasta is a staple food with a low glycemic index (GI), as it progressively releases sugars during digestion, thus leading to low postprandial blood glucose and low insulin response. Traditional pasta is a protein-rich food with unique quality properties, being made by durum wheat semolina [2]. It represents an excellent model food vehicle of specific nutrients through incorporation of various additional ingredients (eggs, milk powder, vegetables, fibers) [3,4].

Lentil, a type of pulse, is a dried seed within the legume family, with great potential to increase satiety, due to good nutritional composition. Lentils are an excellent source of proteins, dietary fibers, oligosaccharides, and starch. Lentil contains bioactive molecules, such as polyphenols, fatty acids, and antioxidants [5], and resistant and digestible starches, protein complexes and prebiotics [6]. Polyphenols isolated from different varieties of lentils showed in vitro inhibition of α-glucosidase, the intestinal enzyme which releases glucose from larger carbohydrates. This specific activity could provide an explanation of low post-prandial blood glucose response, with consequent low GI, associated with the consumption of lentils. Dan Ramdath [7] and Moravek [8] found that replacing half of the available carbohydrates from a starch-rich food with lentils significantly reduced postprandial blood glucose and relative GI in healthy adults. The high content of resistant starch escapes absorption in the small intestine and positively modulates the absorption of carbohydrates and lipids (including cholesterol). Moreover, resistant starch and its anaerobic fermentation derivatives represent a valid growth substrate for the physiological bacteria of the intestinal flora, thus acquiring a remarkable prebiotic function. An increase in the saturated aliphatic organic acid content, together with a reduction in the level of cholesterol and postprandial glucose in the blood, allow associating lentil consumption with lower risk of coronary heart disease [6,9], type 2 diabetes, as well as improved glycemic control and lower blood pressure [10,11], when compared to consumption of animal proteins.

These properties provide a strong rationale for the use of lentil in new fortified pasta. The literature reports data dealing with pasta enriched with lentil flours [12] but these studies are limited to pasta production and analysis of the main quality parameters. To the best of our knowledge, no information is reported on the chemical properties of pasta fortified with lentil flour. Literature only reports that the addition of legume flour to durum wheat semolina can adversely alter pasta tenacity, with negative effects on the starch reticule and, therefore, on pasta water absorption, swelling index, optimal cooking time, consistency, etc. [13,14]. To solve these disadvantages, hydrocolloids as carboxymethyl cellulose (CMC) are generally used because they are able to impart the required quality in terms of stability, texture, and appearance [15,16].

The great industry interest in new types of enriched pasta and lentils represents a good option due to their nutritional properties. In Sicily, different populations of lentils are spread with good variability for some nutritional characteristics, such as protein, fiber, total sugars, and potassium content, mainly due to the climate conditions where they are grown and well adapted (near Etna Mountain or coastal areas). Thus, in the current study two new examples of fortified pasta were produced by adding two lentils of different origin, a commercial variety and a Sicilian local population. To this aim, pasta produced in a pilot plant was studied for technological, sensory, and chemical quality.

## 2. Materials and Methods

### 2.1. Raw Materials

Durum wheat semolina (*Triticum durum* Desf.) was bought from Agostini mill (Montefiore dell’Aso, Ascoli Piceno, Italy) in 2017. The lentil flours (*Lens culinaris* Medik.) were produced using seeds of a commercial lentil cv. Easton (purchased in a local market) and seeds of Sicilian population lentil (population Ragusa, belonging to the CNR ISAFOM in situ germplasm bank, collected during June 2017). Lentils were washed with tap water and dried at low temperature in a thermo-ventilated oven (30 °C) and then milled with a food processor (IKA^®^-Werke GmbH and Co. KG, Staufen, Germany) in fine powder (about 300 micron) and kept in hermetic bottle until use.

### 2.2. Pasta Production and Cooking Procedure

Dry spaghetti samples were produced according to Padalino et al. [16] by mixing durum wheat semolina with water (30% *w*/*w*) in a rotary shaft mixer (Namad, Rome, Italy) at 25 °C for 20 min, to obtain a uniform dough. Three formulations of pasta were made: a control made of exclusively durum wheat semolina (control) and two enriched spaghetti, where 40% semolina (*w*/*w*) was replaced with the commercial lentil flour (LC) or the Ragusa lentil flour (LR). According to findings of a previous study dealing with hydrocolloids in gluten-free spaghetti [16] 2% (*w*/*w*) CMC (Levanchimica S.r.l, Bari, Italy) was added to the enriched formulation. In order to ensure the solubility of the CMC powder, it was previously dissolved in the water used to hydrate the above formulation. The dough was extruded with a MAC 60VR extruder (Namad, Rome, Italy). Subsequently, pasta produced was dried in a dryer (SG600, Namad, Rome, Italy). Each batch of pasta was performed using 3 kg of flours. Spaghetti (100 g) were cooked in 1 L of water with salt (about 15 g) for about 10 min. Three samples were made for repetitions.

### 2.3. Color Analysis

Instrumental color was measured three times on dry spaghetti before and after cooking with two different Minolta CR-400 Chroma meter devices, as described in previous studies [17,18,19].

### 2.4. Sensory Analysis

Dry spaghetti, after cooking at their optimal cooking time, were examined by a panel consisting of 15 trained panelists, with several years of experience in evaluating pasta before this study. For the evaluation, they were asked to indicate color, homogeneity, break to resistance and odor of uncooked samples and elasticity, firmness, bulkiness, adhesiveness, color, odor and taste of cooked spaghetti. A nine-point scale (1 = “extremely unpleasant”, 9 = extremely pleasant”, and 5 = “threshold of acceptability”) was used to quantify each attribute. On the basis of the above mentioned scale, panelists were also asked to score the overall sensory quality of both cooked and uncooked samples [17,19,20].

### 2.5. Cooking Quality

Fortified samples were analyzed for water absorption, cooking loss, swelling capacity and index according to the AACC approved method and literature [16,21]. The optimal cooking time (OCT) was evaluated every 30 s during cooking by observing the time of disappearance of the core of the spaghetti by squeezing them between two transparent glass slides. The time at which the core completely disappeared was taken as the OCT [21]. The analyses were performed in triplicate and the results were the mean of three measurements.

### 2.6. Chemicals and Standards

All solvents, unless specified, ethyl chloroformate (98%), and NaHCO_3_ were purchased from Merck KGaA, (Darmstadt, Germany). Gallic acid and pyridine were from VWR International B.V. (Roden, The Netherlands). Polytetrafluoroethylene (PTFE) syringe filters (25 mm, 0.45 µm pore size) were supplied by Exacta-Optec Labcenter (Italy). The mix of 37 fatty acid methyl ester (FAMEs) components was from Supelco 47885-U, while heneicosanoic acid methyl ester as the internal standard and the standard (Sigma Aldrich 79248) mixture of 17 amino acids 2.5 mM in 0.1 M HCl were from Sigma Aldrich (Milan, Italy).

### 2.7. Fatty Acid Content

A method for selectively determining fatty acids was investigated and optimized using gas chromatography-mass spectrometry (GC/MS) ISQ™ 9000 Quadrupole GC-MS System (Thermo Fisher Scientific, Waltham, MA, USA) after esterification of the target species to their corresponding FAMEs, as described in Melilli et al. [17,19].

A sample (0.5 g) was mixed with 2 mL CHCl_3_ and 1 mL MeOH; the mixture was sonicated for 45 min at 50 °C and cooled to room temperature; it was centrifuged at 4000 rpm for 5 min at 4 °C. The supernatant was filtered with a 0.45 micron PTFE syringe filter and evaporated to dryness under a gentle stream of nitrogen. The obtained residue was dissolved in 100 μL of toluene and 200 μL of KOH in MeOH (10% *w*/*v*). The samples, after 5 min vortex, were added with 200 μL water and 1 mL hexane. The upper phase was collected and 500 μL were added to 200 μL of Internal standard (heneicosanoic acid methyl ester 50 μg/mL) and 300 μL of hexane prior to GC/MS analysis Analyses were made in triplicate, the results are the mean of three measurements, and FAMEs were expressed in mg g^−1^. Total lipids were determined by the Soxhlet method.

### 2.8. Amino Acids (AAs) Quantification by GC-MS Analysis

The modified procedure employed acid hydrolysis of protein and derivatization of the free amino acids using ethyl chloroformate [22].

Fifty milligrams of dry spaghetti sample, shredded with a household grinder, were added with 200 μL HCl 6M. The solution was subjected to nitrogen flow to eliminate the presence of oxygen and to favour the subsequent hydrolysis of proteins and placed in an oven for 24 h at 110 °C to carry out the protein hydrolysis. After cooling to room temperature, the sample has left under nitrogen flow. To the residue, 300 μL water and 300 μL of chloroform were added to the residue. After phase separation, 50 µL of aqueous supernatant were placed in an insert vial with 50 μL ethanol and pyridine solution (4:1 *v*/*v*), 10 μL of ethyl chloroformate (97%), 50 μL of a 1% ethyl chloroformate in chloroformic solution and finally neutralized with 20 μL of a saturated aqueous solution of NaHCO3. The lower phase (AAs derivatized with ethyl chloroformate) was ready for analysis in GC-MS. Derivatized AAs have been identified and quantified through an ISQ™ 9000 Quadrupole GC-MS System (Thermo Fisher Scientific) with ZB -WAX (30 m × 0.25 mm i.d., 0.25 μm film thickness (Phenomenex, Italy). The column was set at 120 °C, increased at 10 °C/min to 240 °C, and held for 3 min under isothermal condition; a second gradient was finally applied to 260 °C with a ramp of 20 °C/min and held for 10 min under isothermal condition. The different AAs were identified on the basis of the characteristic EI(+) fragments that allowed their identification. For this purpose, a standard mixture of AAs (Sigma Aldrich 79248, 17 AA-2.5 mM) was subjected to the same derivatization reaction and injected under the described conditions. Quantitative determination of AAs was performed using calibration curves. Standard solutions of AAs at five concentration levels, in a range from 0.4 mM to 0.025 mM were prepared. Total nitrogen (N) was determined by Kjeldahl apparatus (Büchi, Germany). Proteins were determined multiplying N × 6.25.

### 2.9. Total Phenol Content

The total phenol content in the fortified samples (TPC) was determined using the Folin-Ciocalteu method as reported in literature by Singleton, et al. [23], with some modifications [17,19]. Samples (0.2 g) with 2.5 mL of 50% aqueous methanol were placed in an ultrasonic bath for 60 min at 60 °C and cooled at room temperature. After centrifugation at 3500 rpm for 5 min on the solid residue a further extraction was carried out with 2.5 mL of 50% aqueous methanol. The two aliquots of supernatant were combined. 100 µL of supernatant with 500 µL of Folin-Ciocalteau reagent and 400 µL of 7.5% (*w*/*v*) Na_2_CO_3_ were incubated for 1 h at room temperature in the dark. 150 µL of the solution are inserted on a 96-well plate of a UV-VIS ThermoMultiskan Sky Microplate spectrophotometer (Thermo Fisher) at 750 nm. The calibration curve (y = 5.0944x + 0.1489; R² = 0.9904) was made using eight gallic acid solutions in the range 0.001–0.5 mg/mL. Three replicates of each sample were loaded. The results are expressed as mg gallic acid equivalent per g of dry spaghetti (mg GAE g^−1^).

### 2.10. Data Analysis

Data were submitted to the Bartlett’s test for the homogeneity of variance and then analyzed using one or two-way analysis of variance (ANOVA). Means were statistically separated on the basis of the Student-Newmann-Kewls test, when the ‘F’ test of ANOVA for treatment was significant at least at the 0.05 probability (CoHort Software, CoStat version 6.451).

## 3. Results

### 3.1. Pasta Technological Quality

Addition of lentil flour (40% *w*/*w*) significantly affected the sensory attributes of dry spaghetti (Table 1). No substantial differences were recorded between the two lentil flours, therefore, in the table the sole mean value between the two fortified samples is reported. As regard the uncooked spaghetti enriched pasta recorded an overall quality lower than the control, although it remained acceptable (5.99 vs. 7.50). Specifically, the addition of lentil flours caused a decline in both break to resistance (5.75 vs. 7.00) and color (5.98 vs. 7.50). In general, pasta yellowness decreased when legumes were added, probably due to the lower carotenoid pigments present in the legumes and the existence of other pigments, as pro-anthocyanidins [24]. Concerning cooked spaghetti, the control recorded the greatest overall quality mainly in terms of elasticity, firmness, and adhesiveness (Table 1). The firmness increased in the enriched pasta due to the fact that the lentil flour generally contains more proteins than cereal-based flour [16]. Most probably, the enrichment in protein content and/or the lower water uptake of cooked legume-fortified pasta may be responsible for the higher hardness [16]. However, the incorporation of non-gluten flours diluted the gluten strength and probably weakened the overall structure of the spaghetti. The different chemical composition of durum wheat and legume flour, especially in fiber and protein content, may affect the gluten protein network development, thus giving an enriched spaghetti with increased adhesiveness. The results of CMC hydrocolloid addition to the fortified samples are also shown in Table 1. The addition of CMC significantly changed the sensory parameters of uncooked product, but its positive effect was clearly visible on cooked spaghetti. In general, this behavior can be due to the chemical groups of hydrocolloids that are capable to form hydrogen bonds. This structure has more affinity to starch and forms a stable polymeric network, important for the entrapment of carbohydrates and good pasta quality. Additionally, Chillo et al. [25] found that the presence of CMC slowed down diffusion of amylose molecules from the internal part to the spaghetti surface. Concerning color, taste, and odor, no significant differences among samples with and without hydrocolloid were found.

As regards cooking quality, the replacement of durum wheat semolina with both types of lentil flours in spaghetti statistically reduced (*p* < 0.05) the optimal cooking time compared to the control sample (Table 1). This reduction is probably due to the high dietary fiber contribution that may have facilitated the penetration of water into the pasta core [25]. Loss during cooking increased with the substitution of wheat semolina, as shown by Carini et al. [26] in a pasta-soy formulation. Durum wheat proteins are mainly composed of insoluble proteins (glutenins and gliadins) that are responsible for the formation of intra- and inter-molecular disulfide bonds during processing of dough, thus leading to a strong tridimensional gluten network having the ability to entrap starch granules. On the other hand, legumes are essentially composed of soluble proteins (globulins and albumins) [27]. Thus, the increase of cooking loss in enriched pasta may be due to the introduction of non-gluten proteins that diluted and weakened the gluten network strength.

Data in Table 1 also highlight that enriched spaghetti also recorded a significant rise in water absorption and swelling index, as compared to the control, most probably due to the high dietary fiber content with strong water-absorbing capacity [27,28]. Disruption in the protein matrix would promote water absorption and exposed starch granules to swelling and rupture [20,29]. In the case under study, the 2% CMC incorporation led to an improvement of pasta quality; probably, lentil flour has reduced tenacity, stability, and strength of wheat dough and have diluted gluten. It is also presumable that lentil protein competes with gluten protein for water, thus delaying the process of hydration [30,31]. Table 1 also documents the effects of the incorporation of CMC hydrocolloid on cooking quality. Samples with CMC recorded a rise in OCT value, as compared to the sample without hydrocolloid, most probably due to a lesser water diffusion through the food matrix, which increased the time that water needs to reach the pasta core during cooking [16]. The samples with CMC showed a drop in cooking loss, swelling index and water absorption [32]. With high probability, the CMC forms a network around the starch granules, encapsulating them during cooking and restricting excessive swelling and diffusion of the amylose content, with a consequently greater capacity to absorb and retain water within the very well developed starch-protein-polysaccharide reticule [32].

As regards pasta color evaluated by the colorimeter, the grains of the Ragusa lentil presented L* index higher than the cv. Easton and so pasta samples enriched with this Sicilian lentil had L* values higher than pasta enriched with commercial lentil flour (data not shown). Considering the average for lentil types, L* of enriched spaghetti decreased if compared to the control (Table 1). Using CMC, the brightness of cooked pasta significantly increased (50.0 vs. 47.3). In general, the colorimetric coordinates (L*, a*, and b*) decreased in fortified spaghetti and the results of cooked samples vs. the control indicated the ability of CMC fortified pasta matrix to retain the chromatic components of the lentil flours, especially in terms of b*, which is related to the yellow index of durum wheat pasta.

### 3.2. Pasta Chemical Quality

The comparison between uncooked and cooked control samples showed the presence of unsaturated FAs, in particular ω-6 (linoleic), ω-3 (α-linolenic) and low percentage of saturated FAs (Table 2). The FA content of the two types of lentil flours is very similar; the fatty acid present in greater quantity is the linoleic acid with a relative percentage around 42%, followed by oleic 30% and α-linolenic 10%. In pasta samples, the control showed a fatty acid profile similar to that of the two types of lentil flours, with relative percentages of 43%, 22%, and 5%, respectively. For lentil fortified pasta, the FA profile remains similar for both types of added flours. The linoleic acid with a relative percentage of 44% as the most abundant, is followed by palmitic acid (24%), oleic acid (21%), α-linolenic acid (5%), and stearic acid (4%). The cooking process reduced by about 40% FAs content samples under study (Table 2). In the uncooked spaghetti, the use of CMC added to both types of lentil did not change the content of FAs, compared to the same samples without CMC.

**Figure 1:**
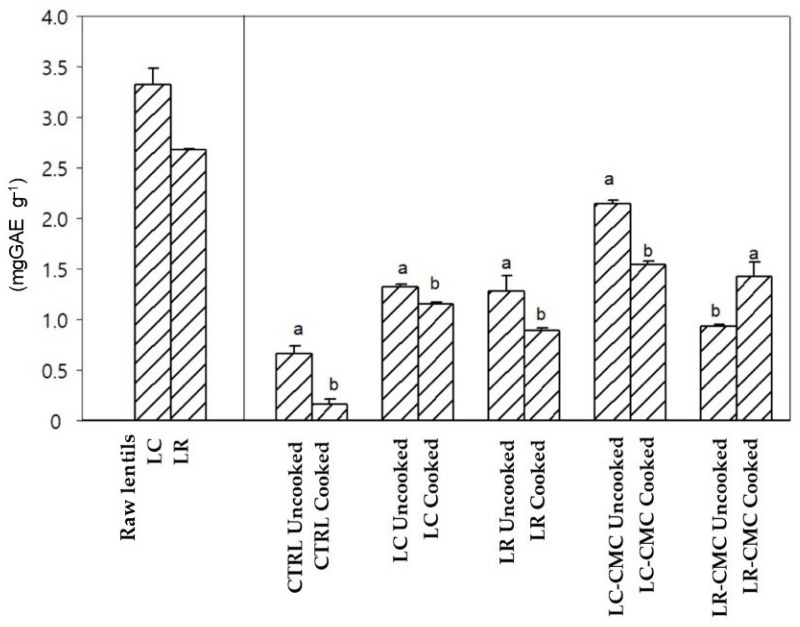
Total phenol content TPC (mg GAE g^−1^) in raw lentils and in uncooked and cooked spaghetti samples with and without lentils of the two accessions, LC and LR; LC-CMC and LR-CMC fortified spaghetti with 2% CMC; *n* = 3. Different letters indicate differences between the two lentil accessions and between uncooked and cooked spaghetti within the same sample. LC, Lentil flour; LR, Ragusa lentil flour; CMC, carboxymethyl cellulose.

As can be seen in Figure 1, total phenol content (TPC), determined in the commercial lentil was 3.32 mg GAE g^−1^, while in the Ragusa lentil was 2.68 mg GAE g^−1^. Comparing the uncooked samples, the total polyphenols ranged from 0.66 (control) to 1.30 mg GAE g^−1^ (LC/LR). Without the hydrocolloid the TPC content has been reduced by 20% compared to uncooked samples. After cooking, a reduction in TPC accounting for about 28% was shown in all the pasta samples. Specifically, TPC for the uncooked LC-CMC was 2.14 mg GAE g^−1^ while it was 1.42 mg GAE g^−1^ after cooking. For LR-CMC the TPC was 1.42 mg GAE g^−1^ for the uncooked pasta and 0.93 mg GAE g^−1^ after cooking.

As far as the protein content is concerned, it is well known that wheat protein is considered to be of poor quality, primarily because it has insufficient amounts of two essential AAs: lysine and threonine [33,34]. Conversely, legumes are a poor source of methionine. These inadequacies in nutritional value of major proteins can be solved combining different protein sources, as suggested in the current study. AAs analysis by GC-MS was carried out in the positive-ion EI mode. Under these conditions, the AAs derivatives undergo extensive fragmentation. The characteristic fragment ions used in single ion monitoring SIM, calibration curve equations and r^2^ coefficient of single amino acids for their quantification are summarized in Table 3.

The observed results showed high concentrations of glutamic acid in a range of 19.7–46.1 mg g^−1^ in uncooked fortified spaghetti. The concentrations dropped in the cooked fortified samples and were in a range between 8.75–21.0 mg g^−1^, higher than the control (4.3 mg g^−1^).

The AAs composition of pasta samples is given in Table 4. In agreement with previous studies, data in the current study confirm that the addition of lentil proteins to dough can significantly increase lysine content in uncooked samples, from 0.31 mg g^−1^ in the control to 1.28 mg g^−1^ in the LC and 1.72 mg g^−1^ in the LR, and threonine from 0.59 mg g^−1^ in the control to 1.29 mg g^−1^ in the LC and 2.46 mg g^−1^ in the LR (Supplementary Material). In pasta samples with lentil addition the increase in AAs involved both essential (EAA) and branched-chain AAs (BCAA). The most striking feature of recorded data is undoubtedly the overall content of EAAs, which in the cooked control pasta is 1.94 mg g^−1^ and in the fortified samples ranges between 7.77 and 9.05 mg g^−1^. Looking at the data of Table 4, it is also possible to infer that in the cooked spaghetti with CMC containing both types of lentil flours increased the contents of EAA and BCAA respect to the control pasta. Moreover, the highest content in total EAA and BCAA is reached using the local population of lentil.

The Table 4, in the last column, reports data of amino acids lost after cooking. It is worth noting that in various cases CMC reduced AA loss, thus assessing its positive role in pasta fortification with lentil flour.

## 4. Conclusions

The main aim of the current study was to develop a protein-enriched pasta with an important lentil content. The emphasis of the study, focused on the AA composition, highlighted a fortified pasta with high lysine and threonine contents. Lentil fortified spaghetti showed high values of essential AAs with improvement of the biological value and nutritional benefits. Addition of legume flour, as non-gluten proteins and insoluble fibers, induced a decrease in pasta quality (e.g., higher cooking loss, lower breaking energy). Some quality attributes of fortified pasta were improved by CMC addition, which caused a drop of cooking loss and rise in swelling index and water absorption, due to gluten dilution. Protein-enhanced pasta is suitable for low-carbohydrate diet and provides three-fold more BCAAs than ordinary durum pasta. The lentil local germplasm used in this study was characterized by a higher protein content and gave a final product richer in EAA. Fortified pasta with lentil flour can help maintaining body mass, muscle mass and enable fat loss, also having an important role in the management of liver cirrhosis. Unfortunately, no increase in mono and polyunsaturated fatty acids was observed in fortified spaghetti, due to their loss during cooking. To sum up, fortified spaghetti with high amount of lentil flour can help defining health claim for legumes, improving the nutritional value without affecting sensory and technological quality.

## Figures and Tables

**Table 1:** Sensory data, color parameters and cooking quality of control and fortified cooked spaghetti.

Sample	Control	LC/LR	LC/LR-CMC
Elasticity	7.25 a	5.12 c	5.50 b
Firmness	7.00 a	5.00 c	5.77 b
Bulkiness	6.75 a	5.15 c	6.23 b
Adhesiveness	6.25 a	4.95 b	6.10 a
Color	7.50 a	6.13 b	6.32 b
Odor	8.00 a	6.63 b	6.93 b
Taste	7.25 a	6.25 b	6.58 b
Overall quality	6.87 a	5.10 c	6.13 b
L*	61.1 a	47.3 c	50.0 b
a*	4.06 c	1.70 a	1.11 b
b*	17.7 a	11.7 b	11.6 b
OCT (min)	9.30 a	8.30 c	9.00 b
Cooking loss (%)	6.18 c	8.03 a	6.97 b
Swelling index	1.80 a	1.96 a	1.77 a
Water absorption (%)	142 b	149 a	138 b

Different letters within the same row indicate statistical differences at *p* < 0.05. LC/LR: mean value of spaghetti obtained with durum wheat and lentil flour on average of the two accessions of lentils; LC/LR–CMC: mean value of spaghetti obtained with durum wheat durum, lentil flour, and 2% of CMC on average of the two accessions of lentils. S.D. < 0.1. LC, Lentil flour; LR, Ragusa lentil flour; CMC, carboxymethyl cellulose.

**Table 2:** Fatty acid content (mg g^−1^ ± S.D.) in lentil accessions and in uncooked and cooked spaghetti: Lentil commercial cv. Easton (LC) and Lentil Ragusa (LR).

Sample	Palmitic Acid	Stearic Acid	Oleic Acid	Linoleic Acid	α-Linolenic Acid	Total Lipids
LC	1.19 ± 0.04	0.23 ± 0.01	2.33 ± 0.05	3.35 ± 0.12	0.86 ± 0.05	4.0
LR	0.81 ± 0.05	0.14 ± 0.02	1.59 ± 0.01	2.30 ± 0.05	0.56 ± 0.02	3.9
Uncooked control	1.26 ± 0.02	0.21 ± 0.02	0.99 ± 0.04	2.01 ± 0.09	0.20 ± 0.03	12.0
Uncooked LC	1.10 ± 0.01	0.22 ± 0.03	0.94 ± 0.01	2.00 ± 0.01	0.26 ± 0.01	8.8
Uncooked LR	1.56 ± 0.08	0.20 ± 0.01	1.01 ± 0.05	2.21 ± 0.12	0.43 ± 0.02	8.8
Uncooked LC-CMC	1.05 ± 0.02	0.18 ± 0.01	0.96 ± 0.01	1.95 ± 0.00	0.18 ± 0.01	7.0
Uncooked LR-CMC	1.03 ± 0.12	0.19 ± 0.01	1.12 ± 0.12	2.95 ± 0.19	0.42 ± 0.02	7.0
Cooked control	1.06 ± 0.04	0.20 ± 0.01	0.82 ± 0.04	0.92 ± 0.11	0.15 ± 0.00	9.2
Cooked LC	0.52 ± 0.01	0.10 ± 0.01	0.42 ± 0.01	0.74 ± 0.01	0.19 ± 0.01	5.4
Cooked LR	0.85 ± 0.05	0.16 ± 0.01	0.56 ± 0.01	0.89 ± 0.02	0.28 ± 0.03	5.3
Cooked LC-CMC	0.60 ± 0.01	0.10 ± 0.01	0.55 ± 0.01	0.85 ± 0.00	0.18 ± 0.01	4.6
Cooked LR-CMC	0.83 ± 0.02	0.13 ± 0.01	0.85 ± 0.00	1.38 ± 0.01	0.21 ± 0.04	4.5

LC and LR: spaghetti obtained with durum wheat flour and lentils of the two accessions; LC-CMC and LR-CMC spaghetti obtained with durum wheat durum flour, lentil of the two accessions and 2% CMC; *n* = 3.

**Table 3:** Amino acids with respective retention times, characteristic fragments of their derivatives in EI, calibration curve equations, and correlation coefficient (r^2^). The basic peaks for each individual amino acid are highlighted in bold.

Amino Acid	Retention Time (Min)	Fragments of Derivatized Amino Acid in EI (m/z)	Equation	Correlation Coefficient (r^2^)
Alanine	8.20	**116**; 88;72;70	y = 2× 10^6^x − 3 × 10^7^	0.7899
Valine	8.86	**116**;**144**;101;98;55	y = 1 × 10^6^x − 1 × 10^7^	0.7862
Isoleucine	10.63	**102**;**158**; 130;69;74	y = 919,938x − 3 × 10^7^	0.7901
Leucine	11.06	**102**;**158**;43;58;72	y = 1 × 10^6^x − 4 × 10^6^	0.7901
Glycine	11.30	**102**;74;57;175;129	y = 1 × 10^6^x − 2 × 10^7^	0.7839
Proline	12.80	**142**;70;98;114;215	y = 1 × 10^6^x − 2 × 10^7^	0.7929
Aspartic Acid	19.51	**188**;**142**;74;56;116	y = 342,170x − 9 × 10^6^	0.8086
Threonine	19.80	**129**;146;175;74;101	y= 538,946x − 2 × 10^6^	0.7665
Methionine	21.90	**175**;142;129;61;249;101	y= 413,384x − 2 × 10^6^	0.7513
Glutamic Acid	21.93	**84**;56;157	y = 121,605x − 2 × 10^6^	0.7381
Serina	22.14	**132**;129;175;74;101	y = 209,841x + 46285	0.7759
Phenylalanine	24.16	**176**;56;84;102;192;91	y = 680,932x − 900,603	0.7693
Arginine	32.01	**156**;56;84;226;128;45	y = 195,53x + 0.03246	0.8503
Lysine	34.86	**149**;167;45;89	y = 680,932x − 900,603	0.8174
Tyrosine	37.44	**107**;192;264;74;164	y = 61,402x + 2 × 10^8^	0.8049

**Table 4:** Total proteins and amino acid content (mg g^−1^) in raw lentils and cooked pasta, as well as AA cooking loss (%).

	Raw Lentils		Cooked Spaghetti					AA Cooking Loss		
	LC	LR	control	LC	LR	LC-CMC	LR-CMC	control	LC/LR	LC/LR-CMC
Alanine	0.30 a ^§^	0.17 b	0.07 c ^§§^	0.36 a	0.36 a	0.28 b	0.25 b	61.12a ^§§§^	56.22 b	46.92 c
Valine *	0.65 a	0.34 b	0.20 c	1.16 a	1.11 a	0.60 b	0.82 b	63.15 a	11.93 c	32.78 b
Isoleucine *	0.64 a	0.34 b	0.18 c	1.12 a	1.06 a	0.55 b	0.79 b	62.46 a	37.57 b	29.98 c
Leucine	1.08 a	0.66 b	0.43 d	2.03 a	1.87 a	0.92 c	1.41 b	57.79 a	31.30 b	31.77 b
Glycine	0.34 a	0.20 b	0.10 c	0.32 b	0.55 a	0.11 c	0.38 b	62.41 a	50.42 b	34.00 c
Proline	0.53 a	0.30 b	0.64 c	2.14 a	1.89 a	1.23 b	1.20 b	46.67 a	40.38 a	45.22 a
Aspartic acid	0.62 a	0.50 a	0.26 c	1.29 b	1.83 a	1.20 b	1.23 b	53.50 a	49.18 b	24.86 c
Threonine *	0.47 a	0.33 a	0.15 b	0.84 a	0.92 a	0.35 b	0.81 a	74.16 a	53.01 b	23.21 c
Methionine *	0.35 a	0.18 a	0.36 b	1.11 a	0.12 b	0.32 b	0.18 b	60.73 a	47.39 b	69.01 a
Glutamic acid	8.8 a	7.2 a	4.30 d	13.2 b	21.0 a	8.75 c	11.5 b	59.04 a	48.01 b	56.62 a
Serina	0.98 a	0.67 b	0.40 c	1.48 b	2.06 a	0.53 c	1.34 b	66.21 a	48.26 b	33.41 c
Phenylalanine *	2.15 a	1.29 a	0.96 c	2.48 b	5.05 a	1.61 c	3.11 b	55.14 a	39.81 b	35.81 b
Lysine *	0.53 a	0.38 a	0.09 b	1.06 a	0.79 a	0.31 b	0.43 b	70.47 a	38.29 b	20.71 c
Total BCAA	2.37 a	1.34 b	0.81 c	4.31 a	4.04 a	2.07 b	3.02 b	60.09 a	28.91 b	31.53 b
Total EAA	4.79 a	2.86 b	1.94 c	7.77 a	9.05 a	3.74 b	6.14 b	60.88 a	39.16 b	35.97 b
Total Proteins	266	282	90	99	103	107	111			

* Essential amino acids; EAA = essential amino acids, BCAA = branched-chain amino acids (leucine, isoleucine and valine). ^§^ Different letters indicate statistical differences at *p* < 0.05 between the two types of lentils, ^§§^ among the five pasta samples and ^§§§^ among control, LC/LR and LC/LR–CMC. LC and LR: spaghetti obtained with durum wheat flour and lentil of the two accessions of lentil; LC-CMC and LR-CMC spaghetti obtained with durum wheat durum flour, and lentil of the two accessions and 2% CMC. S.D. < 0.1.

## Supplementary Materials

The following are available online at https://www.mdpi.com/2304-8158/10/1/4/s1, Table S1: Amino acids content (mg g^−1^) in uncooked lentil fortified spaghetti
