# Supplementary material for: Lentil Fortified Spaghetti: Technological Properties and Nutritional Characterization
Source: Foods. 2020 Dec 22;10(1):4. doi: 10.3390/foods10010004 (PMC7821932; doi:10.3390/foods10010004)
Supplement: Supplementary file 1 [file foods-10-00004-s001.pdf]

Supplementary material

Table S1: Amino acids content (mg g^{-1}) in uncooked lentil fortified spaghetti

	CTRL	LC	LR	LC-CMC	LR-CMC
Alanine	0.17	0.64	0.99	0.60	0.41
Valine*	0.54	1.38	1.20	1.11	0.99
Isoleucine*	0.47	1.34	2.16	1.05	0.85
Leucine	1.02	2.38	3.31	1.27	2.14
Glycine	0.26	0.58	1.18	0.12	0.62
Proline	1.21	2.69	4.07	2.55	1.88
Aspartic Acid	0.56	3.42	2.72	1.87	1.36
Threonine*	0.59	1.29	2.46	0.52	0.99
Methionine*	0.91	1.27	1.07	0.84	0.76
Glutamic Acid	10.4	19.7	46.1	18.1	28.7
Serina	1.20	1.42	5.41	0.73	2.09
Phenylalanine*	2.14	4.46	8.07	3.59	3.76
Lysine*	0.31	1.28	1.72	0.39	0.54
Total BCAA	2.03	5.10	6.67	3.43	3.98
Total EAA	4.96	11.02	16.68	7.5	7.89

*essential aa; EAA=essential amino acids, BCAA=branched-chain amino acids (leucine, isoleucine and valine).
